# Can primary optimal cytoreduction be predicted in advanced epithelial ovarian cancer preoperatively?

**DOI:** 10.1186/1477-7819-8-11

**Published:** 2010-02-19

**Authors:** Azam-Sadat Mousavi, Marjan Moradi Mazhari, Mitra Modares Guilani, Fatemeh Ghaemmaghami, Nadereh Behtash, Setareh Akhavan

**Affiliations:** 1 Department of Gynecology Oncology, vali-e-asr hospital, Tehran University of Medical Sciences, Keshavarz Blvd, Tehran, 1419733141, Iran

## Abstract

**Introduction:**

Prediction of optimal cytoreduction in patients with advanced epithelial ovarian caner preoperatively.

**Methods:**

Patients with advanced epithelial ovarian cancer who underwent surgery for the first time from Jan. to June 2008 at gynecologic oncology ward of TUMS (Tehran University of Medical Sciences) were eligible for this study. The possibility of predicting primary optimal cytoreduction considering multiple variables was evaluated. Variables were peritoneal carcinomatosis, serum CA125, ascites, pleural effusion, physical status and imaging findings.

Univariate comparisons of patients underwent suboptimal cytoreduction carried out using Fisher's exact test for each of the potential predictors. The wilcoxon rank sum test was used to compare variables between patients with optimal versus suboptimal cytoreduction.

**Results:**

41 patients met study inclusion criteria. Statistically significant association was noted between peritoneal carcinomatosis and suboptimal cytoreduction. There were no statistically significant differences between physical status, pleural effusion, imaging findings, serum CA125 and ascites of individuals with optimal cytoreduction compared to those with suboptimal cytoreduction.

**Conclusions:**

Because of small populations in our study the results are not reproducible in alternate populations. Only the patient who is most unlikely to undergo optimal cytoreduction should be offered neoadjuvant chemotherapy, unless her medical condition renders her unsuitable for primary surgery.

## Introduction

Ovarian cancer is the leading cause of morbidity and mortality among the gynecologic cancers [1]. Epithelial ovarian cancers consist 90% of all ovarian cancers [2]. Stage 3 and 4 (as defined by the staging classification of the International Federation of Gynecology and Obstetrics) consist about 2/3 of cases of epithelial ovarian cancer in the time of diagnosis [1-3]. Advanced epithelial ovarian cancers are currently managed with laparotomy + hysterectomy + bilateral salpingooophorectomy + omentectomy + resection of tumoral mass as completely as possible and then platinum based chemotherapy. Maximal diameter of residual tumor after surgery and before starting chemotherapy is an important determinant of prognosis, this has been shown by all studies about advanced epithelial ovarian cancer [4-6].The definition of optimal surgery has been evolved and it is currently defined as residual tumor less than 1 cm [5].Optimal surgery is associated with both a more favorable response to chemotherapy and prolonged survival [7]. The study of GOG has shown that only if the residual tumor is optimal (less than 1 cm) the survival will prolong[5].The success rate of primary optimal cytoreduction for advanced epithelial ovarian cancers is highly variable, depending upon individual and institutional treatment philosophies and experiences. In centers with a particular interest and experience in cytoreductive surgery, rates of optimal resection are reported in 60-90% of cases [8,9].

It is not possible to do primary optimal debulking for all patients, in these cases primary surgery not only dose not have any benefit but also causes morbidity [10]. The 30-day mortality rate for women undergoing primary surgery for ovarian cancer ranged from 1-3% [11]. Moreover, not performing primary surgery in all cases result in omitting the chance of improved survival for some patients.

Primary debulking in patients with advanced epithelial ovarian cancer has been compared with chemotherapy and interval debulking in different studies. Equal survival has been reported in patients undergoing primary surgery compared to patients undergoing debulking surgery after taking chemotherapy by Onnes et al. [12]. They have reported that optimal debulking was achieved in 42% of patients who treated primarily with chemotherapy in comparison with 29% of patients who underwent primary surgery.

In 1999, Shwartz et al. demonstrated that women who underwent cytoreductive surgery after induction chemotherapy had statistically improved overall survival compared to women who did not undergo surgery[13].One randomized prospective study demonstrated that women undergoing interval cytoreductive surgery had improved both overall and progression-free survival[11].It is supposed that less invasive surgery is required for optimal cytoreduction after neoadjuvant chemotherapy. Ansquer et al. in their study have noticed that the morbidity of cytoreductive surgery after neoadjuvant chemotherapy is less than primary debulking [14]. It is noticeable that by performing primary cytoreductive surgery, surgical staging will be done, sensitivity to chemotherapy will increase, risk of mutation will reduce and general status of patient will improve. Considering these, nowadays primary surgery is the preferred management for patients with advanced epithelial ovarian cancer. In America 95% of patients with advanced epithelial ovarian cancer are treated with primary surgery [15].

Regarding that residual tumor is more than 1 cm in many patients underwent primary surgiery, considering another method in this group of patients seems necessary. Although neoadjuvant chemotherapy and interval cytoreduction sounds to be good management but its indications have not yet determined.

A critical point in order to define indications of neoadjuvant chemotherapy for advanced ovarian cancer is determination of uniform selection criteria that can consistently identify patients with surgically unresectable disease without depriving others from potential advantage associated with an optimal primary resection. Several studies have been done for determining markers which can reliably predict optimal resectability [16-18]. CT-Scan findings [17], serum CA-125[18], pleural effusion[19] and ascites [19,20]have been assessed in different studies in order to predict optimal debulking preoperatively but up to now the predictive performance of clinical parameters(e.g ascites), serum CA125 values and imaging criteria have not demonstrated sufficient accuracy to achieve widespread applicability[13]. Thus further investigation concerning patient selection seems warranted. Therefore, we planed the prospective study for assessing the probability of predicting preoperatively optimal cytoreduction with considering combination of variants (abdominal and pelvic CT-scan or MRI findings - presurgical serum CA_125 level- pleural effusion-ascites and physical status) in patients with advanced epithelial ovarian cancer who were admitted at gynecology oncology ward of the Tehran Vali-e-asr hospital and undergoing primary surgery from Jan. to June 2008.

## Patients and Methods

Approval to conduct this study was obtained from research organization of gynecologic oncology department of Tehran University of Medical Sciences(TUMS). Patients with stage 3 and stage 4 epithelial ovarian cancer underwent primary surgery between Jan. to June 2008 at gynecologic oncology ward of Vali-e-Asr hospital of TUMS were eligible for entering the study.

The possibility of predicting primary optimal cytoreduction considering multiple variables was assessed in this group. Variables were peritoneal carcinomatosis, serum CA125 level, ascites, pleural effusion, physical status and imaging findings.

All surgeries were performed by gynecologic oncologists of TUMS. Optimal cytoreduction was defined as ≤ 1 cm residual disease. All imagings were reported by the professors of radiology of TUMS. Considered imaging parameters included: omental extention, liver involvement, peritoneal involvement and suprarenal adenopathy. Blood samples for measuring serum CA125 levels were taken at the morning.

Physical statusesof patients were defined according to physical status classification of the American society of anesthesiology. In addition we considered optimal and suboptimal cytoreduction. Residual tumor less than 1 cm after surgery was considered as optimal cytoreduction.

Univariate comparisons of the percentage of patients who underwent suboptimal cytoreduction carried out using Fisher's exact test for each of the potential predictors. The wilcoxon rank sum test was used to compare variables between patients with optimal versus suboptimal cytoreduction.

## Results

Forty one patients from patients who were admitted at Vali-e-Asr hospital of TUMS from Jan. to June 2008 met study inclusion criteria. Demographic and clinical data are described in table 1. Seventy-three percent of patients had FIGO (international federation of gynecology and obstetrics staging system) stage 3 disease while 17% of patients had FIGO stage 4 disease. Forty-one percent were optimally cytoreduced to ≤ 1 cm residual disease at the time of primary surgery.

**Table 1:** Clinical Data and Tumor Characteristic Study

Characteristic	Patients	
	***No***.	*%*
Clinical status		
1	27	65.9
2	13	31.7

Pleural effusion		
Positive	7	17
Negative	34	82.9

Bowel resection		
Positive	1	2.4
Negative	39	96.6

Intraperitoneal carcinomatosis		
Positive	22	53.6
Negative	19	46.4

**Imaging findings**		

Omental extension		
Positive	6	14.6
Negative	34	85.4

Liver invlovement		
Positive	5	12.1
Negative	36	87.9

Peritoneal involvement		
Positive	12	29.2
Negative	29	70.8

Suprarenal adenopathy		
Positive	0	0
Negative	41	100

CA-125		
≤ 400	11	27.5
>400	29	72.5

Ascitis		
≤ 1000	22	53.6
>1000	19	46.4

Peritoneal carcinomatosis and suboptimal cytoreduction had statistically significant assosciation. There were no statistically significant differences between physical status, pleural effusion, imaging findings, CA125 serum levels and ascites in patients with optimal cytoreduction compared to those who underwent suboptimal debulking.

Table 2 presents the percentage of patients who underwent suboptimal and optimal debulking for each of 9 considered variables. Optimal debulking was performed for 44.4% of patients with physical status 1(according to classification of American society of anesthesiologist (A.S.A)) and 55.6% of these patients undergoing suboptimal debulking. Patients with A.S.A class2 suboptimally debulked in 76.9% of cases and optimlly debulked in 23.1%.About 85% of patients have pleural effusion were suboptimally debulked while only 14.3% of these patients were optimally debulked. Patients who did not have pleural effusion undergoing optimal cytoreduction in 41.2% and suboptimal cytoreduction in 58.8%.We had only one case of bowel resection which resulted in optimal debulking. Suboptimal debulking was performed in 84.2% of patients with peritoneal carcinomatosis,50% with omental extention,60% with liver involvement,58.3% with peritoneal involvement,63.3%with CA125 ≤ 400 and 59.5% with ascites ≤ 1000 in comparison with optimal cytoreduction undergoing in 15.8%,50%,40%,41.7%,36.4%,45.5%of these groups of patients respectively.

**Table 2:** Univariate Analysis of Predictors of Suboptimal Cytoreduction

	patients				
	
	Optimal Cytoreduction		Suboptimal Cytoreduction		
		
Predictor	**No**.	percent	**No**.	percent	P
Clinical status					
1	12	44.4	15	55.6	1.91
		
2	3	23.1	10	76.9	

Pleural effusion					
Positive	1	14.3	6	85.7	.179
		
Negative	14	41.2	20	58.8	

Peritoneal carcinomatosis					
Positive	3	15.8	16	84.2	.01
		
Negative	12	54.5	10	45.5	

Omental extension					
Positive	3	50	3	50	.460
		
Negative	12	34.3	23	65.7	

Liver involvement					
Positive	2	40	3	60	.866
		
Negative	13	36.1	23	63.9	
	
Peritoneal involvement					
Positive	5	41.7	7	58.3	.664
		
Negative	10	34.5	19	65.5	

Adenopathy					
Positive	0	0	0	0	
		
Negative	15	36.6	26	63.4	

CA-125					
≤ 400	4	36.4	7	63.6	.911
		
>400	10	34.5	19	65.5	

Ascitis					
≤ 1000	10	45.5	12	54.5	.205
		
>1000	5	26.3	14	73.7	

## Discussion

Our current study identifies intraperitoneal carcinomatosis as being the only statistically significant predictor of suboptimal cytoreduction. Table 2 demonstrates P value, positive predictive value and negative predictive value of each of the variables for predicting optimal and suboptimal debulking. There were no statistically significant relationship between considered variables and optimal or suboptimal cytoreduction except to intraperitoneal carcinomatosis.

There is no statistically significant difference between pleural effusions in individuals underwent optimal cytoreduction compared to those with suboptimal cytoreduction. It seems that low number of patients caused this result because the number of patients who were suboptimally cytoreduced is in confidence interval range of those who were optimally cytoreduced.The number of patients in our study is only 41. Considering small sample size of the study, proofing these results demands larger randomized study. We used imaging findings as predictive predictors of suboptimal debulking according to previous studies which had mentioned these factors have predictive value.

To date, the predictive performance of clinical parameters, serum CA-125 threshold values, and radiographic imaging criteria have not demonstrated sufficient accuracy to achieve widespread applicability [13,21-24].

The most common criteria cited as justification for abandoning an up-front attempt at surgical cytoreduction are ascites volume greater than 1000 ml, peritoneal carcinomatosis, parenchymal liver disease, splenic metastasis or omental extension to the spleen, porta hepatitis disease, and bulky disease involving the diaphragm[8] one of the earliest studies attempting to forecast the surgical outcome of patients with advanced stage ovarian cancer assessed the predictive value of these criteria in a series of 42 patients[15].In this sentinel study, Nelson et al reported a positive predictive value for a suboptimal surgical result of 67%.Not to be overlooked, it is the fact that one out of every three patients thought to have unresectable tumor would have been left with optimal residual disease if offered primary surgery. More recently, Axtell et al. [25] reported data that highlight the difficulty in defining universally applicable selection criteria that reliably predict surgical outcome across institutions and surgeons.

One of the principle difficulties in development of any reliable predictive model of surgical outcome for patients with advanced ovarian cancer is the challenge of factors in the significant impact of each institute surgeons' philosophy, effort and ability to utilize advanced surgical techniques to achieve maximal cytoreduction, in order to omit this factor, in this study all surgeries were performed by gynecologic oncology professors of TUMS.

In summary, identification of risk factors for suboptimal cytoreduction in small populations such as ours is not reproducible in alternate populations. Until prospective randomized trials have demonstrated that neoadjuvant chemotherapy followed by interval cytoreduction is equivalent in terms of survival outcomes to primary optimal cytoreduction followed by chemotherapy, extreme caution should be used when applying preoperative predictors to decide between primary surgical exploration and neoadjuvant chemotherapy in the medically fit patient. Only the patient who is most unlikely to undergo optimal cytoreduction should be offered neoadjuvant chemotherapy, unless her medical condition renders her unsuitable for primary surgery.

## Competing interests

The authors declare that they have no competing interests.

## Authors' contributions

AM: supervised research project, carried out operations, supervised statistics. MMM: participated in operation as first aid, collect data, drafted the manuscript, and acted as corresponding author and did the revisions. MMG: carried out operations, she was head of the department. FG: carried out operations. NB: carried out operations. SA: participated in operation as first aid.
